# Effects of Cardiopulmonary Bypass Surgery on Auditory Function: A Preliminary Study

**DOI:** 10.1155/2013/453920

**Published:** 2013-08-29

**Authors:** Sanjay Kumar Munjal, Parul Malik, Anuradha Sharma, Naresh Kumar Panda, Shyam K. Singh Thingnum

**Affiliations:** ^1^Speech and Hearing Unit, ENT Department, New OPD, Postgraduate Institute of Medical Education & Research, 4th floor, Chandigarh 160012, India; ^2^Department of Otolaryngology, Postgraduate Institute of Medical Education & Research, Chandigarh 160012, India; ^3^Department of Cardiothoracic and Vascular Surgery, Postgraduate Institute of Medical Education & Research, Chandigarh 160012, India

## Abstract

Hearing loss has been reported as a complication following cardiac surgery with extracorporeal circulation. Preoperative hearing testing is not commonly done in these procedures, so preoperative and postoperative hearing loss, if any, may occur unnoticed. 30 subjects in the age range of 50–70 with a mean age of 60.16 years with myocardial infarction and scheduled to undergo cardiopulmonary bypass surgery underwent detailed audiological assessment comprising of pure tone audiometry with extended high frequency audiometry, speech audiometry and otoacoustic emissions (OAE) testing. The audiological testing was done preoperatively and at 2 weeks after the surgery. On pure tone audiometry, the difference between pre- and postsurgery mean values for both ears at 10, 12, and 16 KHz showed highly significant differences (*P* < 0.0001). On OAE testing, a significant difference (*P* < 0.05) between pre- and postvalues of signal to noise ratio (SNR) was found. It is hypothesised that CPB surgery makes blood redistribution to other organs easy, deviating from internal ear, which is highly susceptible as it lacks collateral circulation and its cells have high energy metabolism. Epithelial damage on internal ear microcirculation causes reduction of the cochlear potentials and hence hearing loss.

## 1. Introduction

Hearing is one of the most significant of human senses. For an individual, it is perhaps the most crucial link in communication with the outside world. It is a prerequisite for fulfilling life and career. Any deficit in it is as frightening for the person as a loss of vision. It is a handicap potentially severe enough to deny a person a normal life and livelihood [1]. Various etiologies have been proposed to explain the sudden sensorineural hearing loss (SSNHL) in the general population and include inflammatory, vascular, metabolic, traumatic, membrane rupture, functional, or idiopathic [2].

In recent decades, the development of SSNHL with nonotologic surgeries carried out under general anesthesia has become a great area of interest. A considerable majority includes cardiopulmonary bypass procedures, an association that has long been recognised [3].

Cardiopulmonary bypass (CPB) is a technique that temporarily takes over the function of the heart and lungs during surgery, maintaining the circulation of blood and the oxygen content of the body. Cardiopulmonary bypass surgery is a common practise in many medical centres, and instances of unilateral hearing loss in these patients have been reported. The first report of this kind was made by Arenberg et al. [4] who reported sudden unilateral deafness immediately following cardiopulmonary bypass. This was followed by a larger retrospective study by Plasse et al. [5, 6] who found the incidence of SNHL to be 1 in every 1000. Many researchers have postulated a genetic basis behind this sudden development of hearing loss following surgery [7, 8]. Extracorporeal circulation usage seems to bring an additional risk in terms of hearing loss [9].

## 2. Need for the Study

Hearing loss has been reported as a frequent complication following cardiac surgery with extracorporeal circulation. Preoperative hearing testing is not commonly done in these procedures, so preoperative and postoperative hearing loss, if any, may occur unnoticed. 

## 3. Aim of the Study

1. To investigate the differences between pre- and postoperative hearing thresholds, measured by conventional and extended high frequency audiometry.
2. To assess the function of the outer hair cells of the inner ear before and after the surgery with the help of otoacoustic emissions.

## 4. Materials and Methods

### 4.1. Sample

A total of 30 subjects with myocardial infarction scheduled to undergo cardiopulmonary bypass surgery were referred from the Advanced Cardiac Centre (ACC) for detailed audiological assessment to Speech and Hearing Unit, PGIMER, Chandigarh.

A two-channel dual clinical/diagnostic audiometer Madsen Orbiter 922 was used to assess the pure tone thresholds, speech reception thresholds, and speech discrimination score of all the subjects. An immittance audiometer Madsen Zodiac 901 calibrated as per ANSI 1992 standards was used to assess the middle ear function and thus rule out the middle ear pathology. Otoacoustic emissions (OAEs) were measured with IHS instrument to assess the outer hair cell activity and thus find cochlear dysfunction.

### 4.2. Procedure

Air and bone conduction thresholds were obtained for pure tone stimuli in a sound treated room at frequencies ranging from 250 to 4000 Hz measured at each octave interval. Further, high frequency audiometry was performed up to 16 kHz. To evaluate the speech sensitivity, speech audiometry was done. The lowest intensity, at which 50% of the spondee words were repeated correctly, was taken as speech reception threshold (SRT). Once the SRT was measured, 50 phonetically balanced (PB) words in Hindi/Punjabi 40 dB above the SRT were introduced in each ear of the patient. The number of correctly repeated words was noted, and speech discrimination score was computed.

Detailed case history was taken from interviewing of patients for subjective analyses. The auditory function was gauzed pre- and postoperatively for objective assessment. The following clinical parameters of the patients were documented:

1. age,
2. sex,
3. history of diabetes mellitus,
4. history of hypertension,
5. history of myocardial infarction,
6. whether going for CBP surgery,
7. postsurgery effect on hearing.

The first audiogram was done preoperatively one day prior to surgery to assess the baseline level of a subject's hearing. The second audiogram was done 2 weeks after the surgery to assess the alteration in hearing of the subjects. However, to further monitor the cochlear function otoacoustic emissions, namely, distortion product otoacoustic emissions (DPOAEs) and transient product otoacoustic emissions (TOAEs), were recorded. TOAEs were measured using a wide band click in a continuous mode, with an intensity of 90 dB SPL. When measuring DPOAEs, the frequency separation of the primaries was *f*_2_/*f*_1_ = 1.22 and *L*_1_ and *L*_2_ set to 65 and 55 dB SPL, respectively.

## 5. Results

### 5.1. Statistical Methods and Data Analysis Procedures

The data was analysed using appropriate statistical measures. The value of mean for central tendency and standard deviation for variability was computed. Mean values of thresholds and signal to noise ratio, at different frequencies, were compared before and after surgery using paired *t*-test.

### 5.2. Ethical Justification

This study was conducted in the Department of ENT, PGIMER, Chandigarh. The study took care of all the following ethical consideration. Written informed consent was taken from the patients. Subjects were informed about their right to withdraw consent from investigation at any time without jeopardizing their treatment. No invasive procedure was involved as a part of investigation, and these investigations had no side effects.

### 5.3. Observations and Results

In the present study, conventional audiometry, and high frequency audiometry followed by transient and distortion product otoacoustic emissions were conducted in 30 patients before and after cardiopulmonary bypass surgery.

### 5.4. Patient Characteristics

All the subjects for the study were in the age range of 50–70 years with a mean age of 60.16 years in males (SD = 1.9) and 62.23 in females (SD = 2.1).  Table 1 shows the mean age and sex distribution. It is to note that 12 subjects in the male category had history of myocardial infarction and complaints of hypertension and diabetes mellitus. However, the number was 7 in case of female category.

### 5.5. Audiological Findings

Table 2 shows the comparison of pure tone audiometry and speech audiometry of the subjects before and after CPB surgery. PTA1 depicts the average of the air conduction thresholds at 250 Hz, 500 Hz, and 1 kHz. Moreover, PTA2 depicts the average of the same parameter at 2 KHz, 4 KHz, and 8 KHz.

As can be inferred from the previous results, no statistically significant difference (*P* > 0.05) in the pre- and postoperative mean values was found for the parameters mentioned previously (namely, PTA1, SRT, and SDS). However, difference was significant for PTA2 (*P* < 0.001).

Table 3 shows the comparison of mean values of subjects before and after CBP surgery for extended high frequency audiometry. As can be observed, significant difference in the mean thresholds in the high frequency spectrum (10–16 KHz) was found in both ears after CPB surgery.

Table 4 shows the comparison of pre- and postoperative mean SNR values of DPOAESs in subjects undergoing CPB surgery. As can be seen, a significant difference (*P* < 0.05) in the signal to noise ratio was found for the frequencies 704 Hz, 1003 Hz, 2000 Hz, 1409 Hz, and 5649 Hz in the right ear and 704 Hz, 1003 Hz, 2000 Hz, and 5649 Hz in the left ear, respectively.

Table 5 shows the comparison of pre- and postfindings of TEOAES in subjects undergoing CPB surgery. It is clearly observable that significant decrease (*P* < 0.05 to *P* < 0.001) in the signal to noise ratio was found after the CPB surgery in the high frequency spectrum of TEOAEs (2 KHz–4 KHz) in both ears.

The individual data was also analysed based on the change in hearing threshold (in dB) during pure tone audiometry and change in SNR values (in dB) during OAEs testing after CPB surgery.

Table 6 shows the number of subjects with change in thresholds of >15 dB and >30 dB for PTA1, PTA2, and PTA3 after CPB surgery. It was found that 10 subjects had more than 15 dB and 4 had more than 30 dB change in thresholds, respectively, on PTA2. For PTA3, more than 15 dB change was seen in 13 subjects, and 6 subjects had a change in thresholds of more than 30 dB. However, for PTA1, only one subject had change in thresholds of more than 30 dB. Hence, the results revealed that in the majority of the subjects high frequency hearing was affected after CPB surgery.

Table 7 shows the number of subjects with change in SNR values of >1 dB and >2 dB after surgery. 13 subjects had change in SNR of more than 2 dB, and 6 had change of more than 1 dB on TOAEs testing, whereas, on DPOAEs testing, a change of more than 2 dB was observed in 11 subjects and 8 subjects, had a change of 1 dB.

Hence, the findings revealed that 19 out of 30 subjects had changes in cochlear function.

It becomes essential to mention that, while analysing the data, it became clearly evident that the 12 males and 7 females who had history of hypertension and diabetes mellitus formed the majority of the patients who had significant postoperative changes in the auditory function. Furthermore, no significant postoperative changes could be traced in 11 out of total 30 subjects.

## 6. Discussion

Sudden sensorineural hearing loss is a frequent problem being encountered by the audiologist. There are many etiologies including fluctuation in blood pressure, blood sugar, viral infections, and consequences of many surgeries which lead to sudden loss of hearing sensitivity. Cardiopulmonary bypass surgery is one major surgery after which patients have reported hearing loss of varied degrees. However, despite the proposition of diverse hypotheses in the past, the pathophysiology remains unclear. This preliminary study was planned to obtain a better understanding of the effects of cardiopulmonary bypass surgery on the auditory function. The present study was designed to identify the changes in hearing sensitivity in terms of thresholds and outer hair cell activity following cardiopulmonary bypass surgery.

In the present study, there was no significant alteration in the pure tone thresholds in the frequency range from 250 Hz to 8 KHz during air and bone conduction after the CPB surgery. However, the study carried out by Ashraf [1] reported a significant increase in the thresholds even in conventional audiometry (250 Hz–8 KHz) after CBP surgery. Moreover, it also reported that some additional patient parameters like cooccurrence of diabetes mellitus and hypertension may influence this process. These findings are in support of our findings where we noticed that coexisting conditions like hypertension and diabetes mellitus can add up to the auditory dysfunction. The first report of this kind was made by Arenberg et al. [4] who reported sudden unilateral deafness immediately following cardiopulmonary bypass. Earlier, Brownson et al. [10] had tried unsuccessfully to achieve the same in their study with a limited sample. However, these results are highly questionable considering the presence of false negatives and false positives associated with retrospective studies.

In the high frequency audiometry, hearing sensitivity was evaluated in the frequency domains of 10 KHz, 12 KHz and 16 KHz. The results showed highly significant differences between the pre- and postoperative thresholds (*P* < 0.0001) in both ears, in all the high frequencies tested. Furthermore, 6 patients reported considerable hearing loss immediately after surgery. These findings are in well agreement with the reports of Walsted et al. [11] who described 4 cases of hearing loss after CPB surgery. Out of these 4 patients, 3 had hearing loss as soon as they recovered from anaesthesia, while the fourth experienced a loss in hearing over the first week after the surgery. This immediate alteration in the hearing acuity can be attributed to effects of anaesthesia, prolonged concentration of ototoxic drugs in the blood, and reduced blood supply to the ear via microcirculation. Phillipps and Thornton [12] also reported the occurrence of high frequency hearing loss in patients who underwent cardiopulmonary bypass surgery as compared to a control group who underwent thoracotomy but without being placed on a cardiopulmonary bypass. They suggested that the patient's age, minimum temperature, and minimum blood pressure during the operation and the time on bypass were the discriminating factors affecting the hearing. Also, sudden SNHL after CPB surgery has been reported by Evan et al. [13] and was postulated to be due to microemboli generated by atheromatous material. Aytacoglu et al. [9] reported that the risk of unfavorable changes in hearing thresholds was augmented by the use of extracorporeal circulation.

While assessing the cochlear hair cell activity through distortion product and transient evoked otoacoustic emissions, it was found that there was a highly significant decrease in the signal to noise ratio at 2822 Hz, 3991 Hz, and 5649 Hz in both ears while in the left ear at 2000 Hz after the CPB surgery. While documenting TEOAEs, it was found that there was significant decrease in the signal to noise ratio at 2 KHz, 3 KHz, and 4 KHz in both ears after the CPB surgery. Our findings are in well agreement with the findings of Borin and Cruz [14] who evaluated the function of cochlear outer hair-cells under the influence of extracorporeal circulation and moderate hypothermia during cardiac surgery. They found that the amplitudes of DPOAEs decreased during moderate hypothermia induced during extra-corporeal circulation. The possible hypothesis behind this hearing loss following CPB surgery is that the surgery makes blood redistribution to other organs easy, deviating from internal ear, which is highly susceptible as it lacks collateral circulation and its cells have high energy metabolism. Epithelial damage on internal ear microcirculation causes reduction of the cochlear potentials. The damages produced in internal ear microcirculation during general anaesthesia lead to a lack of oxygen of the ciliated cells, and hence, hearing loss.

## 7. Conclusions

 This preliminary study shows that open heart surgery using cardiopulmonary bypass can lead to significant postoperative changes in hearing levels at some frequencies. These changes can be associated or may be due to reduced cochlear activity as estimated by OAEs. Some additional patient parameters like the presence of hypertension, diabetes mellitus, and history of myocardial infarction may influence this process. Further research is needed to see if this induced dysfunction can be controlled, which in turn will reduce the risk of developing hearing loss after surgery. These are the findings of preliminary work on small sample. We are planning to extend this study on a large sample wherein the subjects would be followed up for a period of one year to monitor the hearing status and to detect the delayed onset of hearing loss, if any.

 Based on the present findings, it is suggested that every patient undergoing cardiopulmonary bypass surgery must be evaluated preoperatively and postoperatively with the help of audiological battery (conventional and high frequency audiometry and otoacoustic emissions), so that hearing loss, if any developed, can be detected and managed at the earliest time.

## Figures and Tables

**Table 1:** Mean age and sex distribution.

Sex	Number of subjects	Percentage (%)	Mean age (yrs)
Males	18	60	60.16
Females	12	40	62.23

**Table 2:** Comparison between pre- and postoperative mean values on pure tone audiometry.

	Right ear				*t* value	Left ear				*t* value
	Mean (before)	Mean (after)	SD (before)	SD (after)		Mean (before)	Mean (after)	SD (before)	SD (after)	
PTA1 (dB HL)	24.44	24.33	5.59	4.96	0.44	24.11	24.22	5.34	4.95	0.53
PTA2 (dB HL)	31.78	32.94	6.12	5.46	4.58***	31.28	32.83	6.18	6.18	4.47***
SRT (dB HL)	27.12	27.29	4.56	4.91	0.43	27.34	27.54	4.78	4.92	0.77
SDS (%)	88.13	88.26	3.9	3.87	0.21	89.12	89.09	2.86	3.86	0.27

*P < 0.05, **P < 0.01, and ***P < 0.001.

**Table 3:** Comparison of the mean values of extended high frequency audiometry in the subjects before and after CBP surgery.

Threshold	Right ear				*t* value	Left ear				*t* value
	Mean (before)	SD	Mean (after)	SD		Mean (before)	SD	Mean (after)	SD	
10 kHz	37.00	5.81	39.00	6.49	3.52**	36.83	6.23	38.50	6.45	3.34**
12 kHz	40.17	7.01	42.83	8.06	3.76**	39.67	6.81	43.33	6.99	5.12***
16 kHz	43.50	7.33	47.17	8.06	6.28***	42.83	6.78	48.17	8.25	3.79***

*P < 0.05,**P < 0.01, and ***P < 0.001.

**Table 4:** Comparison of the mean SNR values of DPOAEs in the subjects before and after CBP surgery.

Frequency	Right ear				*t* value	Left ear				*t* value
	Mean (before)	SD	Mean (after)	SD		Mean (before)	SD	Mean (after)	SD	
357 Hz	5.23	3.5	4.98	3.12	1.03	5.07	3.48	4.80	3.24	1.97
499 Hz	5.30	3.56	4.87	2.91	1.37	5.5	3.60	5.45	3.52	1.31
704 Hz	3.87	3.87	3.17	2.79	2.17*	4.30	3.65	3.70	3.11	2.34*
1003 Hz	3.47	3.31	2.63	2.71	2.62*	3.60	3.00	3.33	3.10	0.81*
1409 Hz	2.23	3.08	1.80	3.25	1.06*	2.27	3.88	1.80	3.25	1.88
2000 Hz	1.03	2.85	0.33	2.81	1.21*	1.10	2.54	0.79	2.77	2.11*
2822 Hz	−0.60	2.87	−0.73	2.93	0.90	−0.87	3.77	−1.23	4.13	1.38
3991 Hz	−1.30	3.34	−1.50	3.50	0.69	−1.31	3.30	−1.51	3.48	0.71
5649 Hz	−2.30	3.78	−2.80	3.84	2.01*	−2.00	3.73	−2.67	3.84	2.21*

*P < 0.05, **P < 0.01, and ***P < 0.001.

**Table 5:** Comparison of the mean SNR values of TEOAEs in the subjects before and after CBP surgery.

	Right ear				*t* value	Left ear				*t* value
	Mean (before)	SD	Mean (after)	SD		Mean (before)	SD	Mean (after)	SD	
1 KHz	4.11	2.15	4.18	2.12	0.48	4.49	1.95	4.47	1.91	0.12
1.5 KHz	3.43	2.34	3.25	2.24	1.32	2.87	2.01	3.11	2.23	1.31
2 KHz	0.89	4.19	0.52	3.99	2.05*	1.00	4.07	0.33	4.06	2.61*
3 KHz	−0.30	3.81	−1.00	3.74	3.32**	−0.20	3.98	−0.99	3.74	2.41*
4 KHz	−2.73	3.07	−3.48	3.01	3.16**	−2.83	3.46	−3.83	3.76	3.94***

*P < 0.05, **P < 0.01, and ***P < 0.001.

**Table 6:** Postoperation changes in hearing thresholds.

	PTA1 (250, 500, and 1000 Hz)		PTA2 (2 K,4 K, and 8 KHz)		PTA3 (10 K,12 K, and 16 KHz)	
Change in hearing thresholds	>15 dB	>30 dB	>15 dB	>30 dB	>15 dB	>30 dB
Number of subjects	0	1	10	4	13	6

**Table 7:** Postoperation changes in OAEs.

Type of OAEs	TOAEs		DPOAEs	
Change in SNR at 3 consecutive frequencies	>2 dB	>1 dB	>2 dB	>1 dB
Number of subjects	13	6	11	8
